# The “appearing” and “disappearing” ascites in the treatment of colorectal cancer: a case report

**DOI:** 10.3389/fonc.2024.1372812

**Published:** 2024-06-27

**Authors:** Hong-Ming Cui, Xin-Peng Shu, Zheng-Qiang Wei, Xing-Ye Wu

**Affiliations:** Department of Gastrointestinal Surgery, The First Affiliated Hospital of Chongqing Medical University, Chongqing, China

**Keywords:** colorectal cancer, chemotherapy, ascites, oxaliplatin, case report

## Abstract

**Background:**

Colorectal cancer (CRC) is one of the most common cancers worldwide. In the treatment of patients with CRC, oxaliplatin plays a pivotal role, with moderate side effects. Neurotoxicity, myelosuppression, ototoxicity, delayed hypersensitivity reactions, and rhabdomyolysis induced by oxaliplatin have been reported individually. However, the occurrence of oxaliplatin-induced ascites has not been reported previously. The objectives of this case report were to elaborate on the rare occurrence of ascites in a patient with CRC after oxaliplatin therapy and to explore its characteristics and causes.

**Case description:**

We report on a case of upper rectal cancer seen in a 65-year-old man who underwent robotic-assisted laparoscopic anterior rectal resection. The patient developed ascites during postoperative adjuvant therapy with oxaliplatin and capecitabine. We ruled out tumor recurrence by laparoscopy, intraoperative biopsy, and biochemistry of the ascites. The patient did not experience a recurrence of ascites after discontinuation of chemotherapy.

**Conclusion:**

This case suggests that chemotherapy with oxaliplatin might cause ascites. The mechanism of the oxaliplatin-induced liver injury was further discussed, which might have been the cause of ascite formation. When patients with CRC who underwent chemotherapy with oxaliplatin develop ascites, surgeons should actively determine whether this is a side effect of chemotherapy or is due to tumor recurrence in order to avoid unnecessary surgery.

## Introduction

According to GLOBOCAN 2020, colorectal cancer (CRC) is third in terms of incidence among cancers all over the world. Moreover, CRC has the second highest global cancer mortality rate, with 935,000 deaths per year (1). Although the incidence and mortality of CRC are high, patients with advanced CRC have better prognosis. The common diagnostic methods for rectal cancer include colonoscopy, computed tomography (CT) scan, and magnetic resonance imaging (MRI). These methods can not only accurately assess the size, location, and depth of tumor invasion but also provide an important basis for the development of personalized treatment. In addition to the increasing sophistication of surgical techniques, great progress has relied heavily on the advances in chemotherapy (2). Moreover, targeted therapy, immunotherapy, and radiotherapy have also been applied in CRC.

The chemotherapy for CRC is based on fluorouracil or its analogs. The combination of oxaliplatin and fluorouracil has a good synergistic effect and is also frequently used in chemotherapy for CRC, with good results (3). Oxaliplatin targets DNA as the site of action, and its platinum atoms form cross-links with DNA, which could antagonize DNA replication and transcription (4).

Although its curative effect is significant, the accompanying adverse reactions cannot be ignored. Cases of ascites induced by oxaliplatin have been reported in the past, but have been mainly attributed to complications of oxaliplatin-induced cirrhosis (5, 6). The occurrence of ascites may be misdiagnosed as tumor recurrence and metastasis. In this context, this article reports the phenomenon of ascites after oxaliplatin treatment in a patient with rectal cancer, aiming to analyze its characteristics and possible mechanisms.

## Case presentation

**Figure 1:**
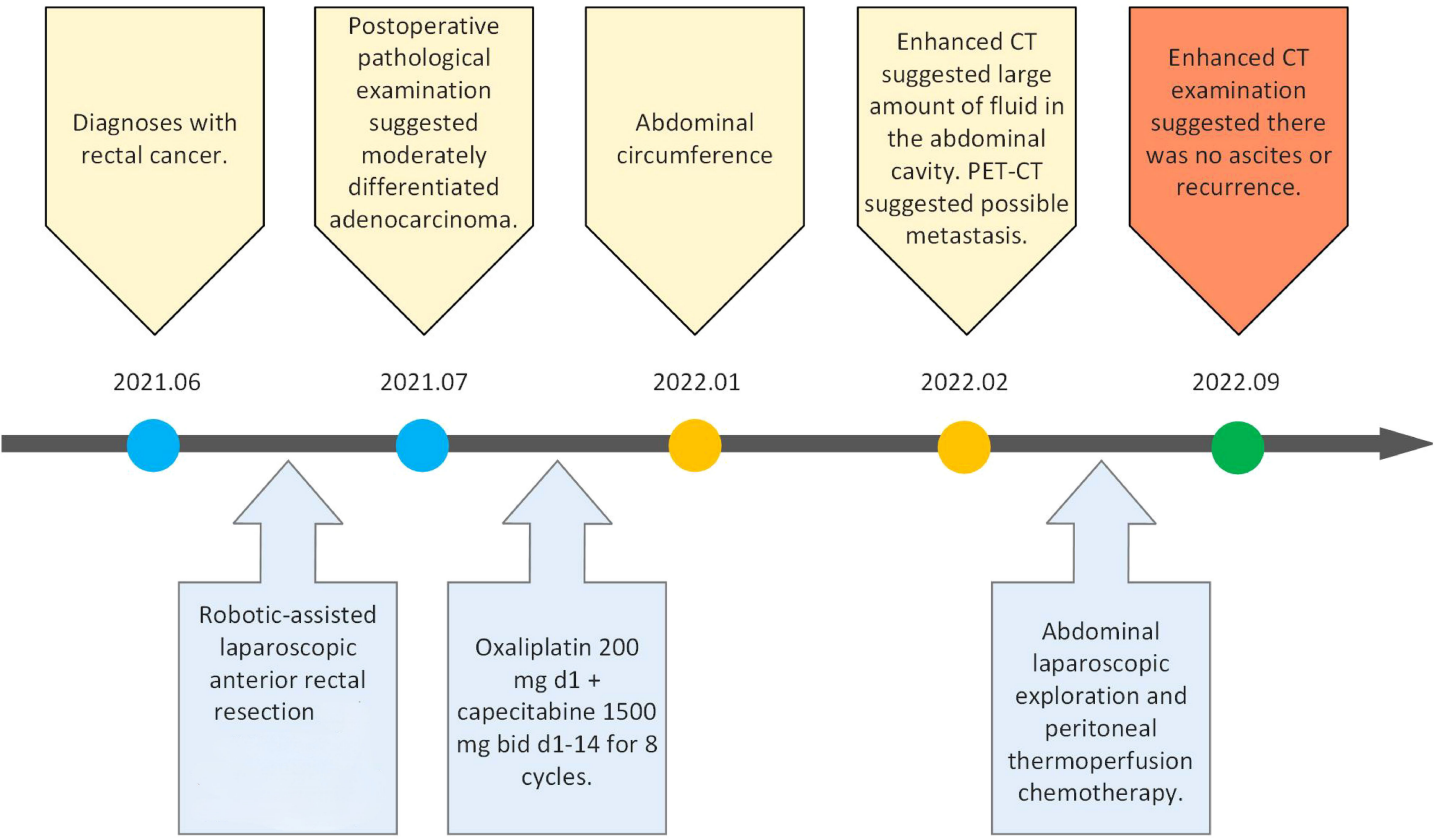
Timeline of patient clinical events.

The timeline of the 65-year-old male patient in this study is shown in **Figure 1**.

### Preoperative examination

**Figure 2:**
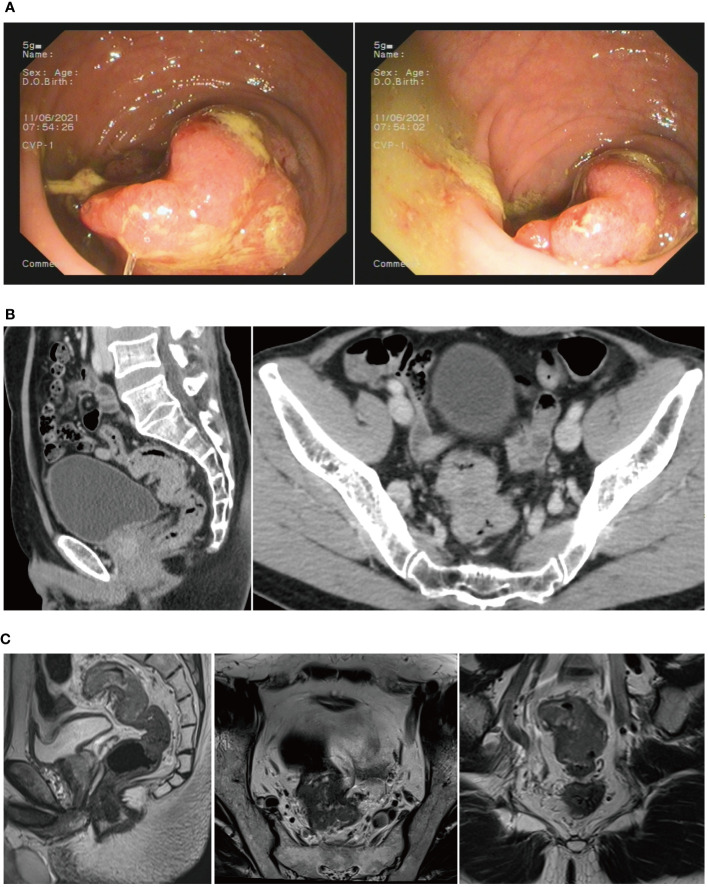
**(A)** Colonoscopy revealed a rectal cauliflower-like neoplasm. **(B)** CT scans showed a tumor-like lesion in the rectum. **(C)** MRI suggested rectal cancer.

The patient was admitted to the First Hospital of Chongqing Medical University with the chief complaint of “change in stool habit for 5 months.” The rectal finger examination did not reveal a mass, but the retracted finger was stained with blood. The colonoscopy on June 11, 2021, suggested the presence of a rectal cauliflower-like neoplasm at 11 cm from the anal verge, with surface erosion and bleeding, invading most of the intestinal lumen so that it was narrowed, only allowing the colonoscope to barely pass through (**Figure 2A**). Pathological examination after colonoscopy suggested adenocarcinoma. Enhanced CT on June 13, 2021, suggested an uneven eccentric thickening of the rectal wall with the plasma membrane surface rough, which was considered as a tumor-like lesion with a high possibility of rectal cancer. The lesion was surrounded by multiple small lymph nodes in the superior rectal artery (**Figure 2B**). Moreover, the pelvic MRI suggested rectal cancer at 11.0 cm from the anal verge, with the MR stage at T4aN1, circumferential resection margin (CRM) (+), extramural venous invasion (EMVI) (−), and enlarged lymph nodes behind the left seminal vesicle (**Figure 2C**). The CA19–9 was 89.9 kU/L. The patient was diagnosed with upper rectal cancer.

### The primary surgery

Surgery was conducted after discussion with the multidisciplinary team (MDT). Robotic-assisted laparoscopic anterior rectal resection was performed on June 17, 2021. Postoperative pathological examination suggested a moderately differentiated adenocarcinoma, which invaded the entire intestinal wall up to the peri-intestinal adipose tissue. No cancer involvement was seen in the bilateral bowel margins and radial margins. Cancer metastasis was seen in the lymph nodes of the peri-intestinal fat (7/18), and another eight cancer nodes were discovered. Immunohistochemistry suggested MSH2(+), MSH6(+), HLH1(+), PMS2(+), BRAF(−), and Ki-67 75%(+). Genetic testing suggested microsatellite stability (MSS), without *KRAS*, *NRAS*, and *BRAF* gene mutations detected.

### Postoperative chemotherapy and ascites “appearing”

After surgery, eight cycles of postoperative chemotherapy [oxaliplatin 200 mg, ivgtt d1 (q3w) + capecitabine 1,500 mg, po bid d1–14 (q3w)] were administered on July 20, August 17, September 7, September 28, October 19, November 16, December 7, and December 28, 2021.

**Figure 3:**
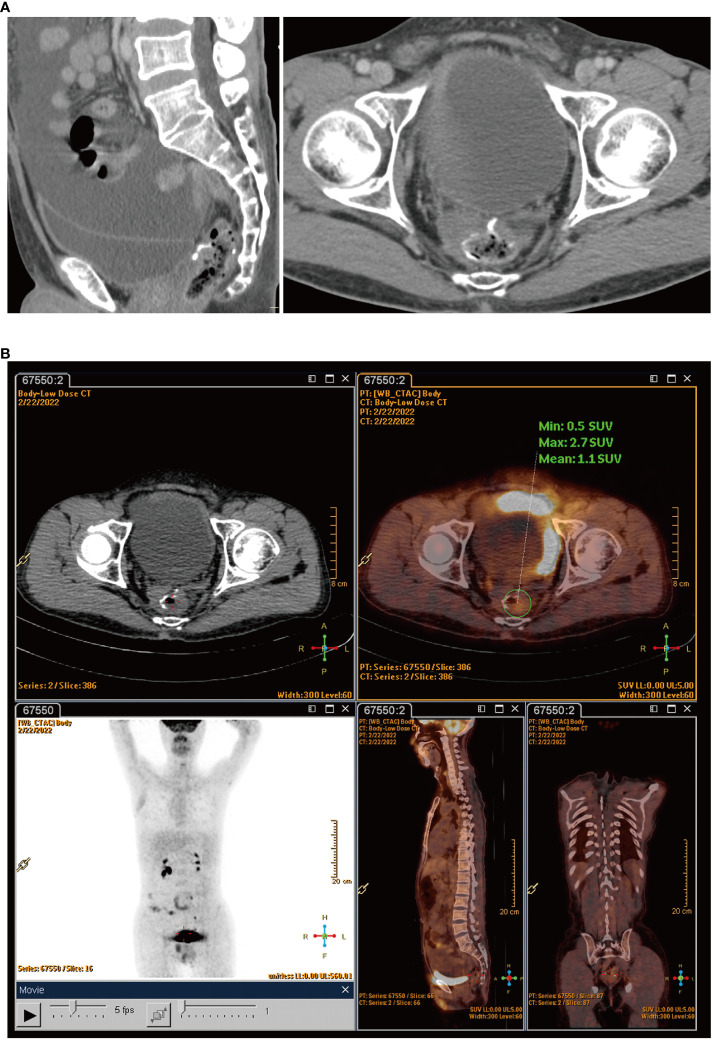
**(A)** Rescan of enhanced CT found a large amount of fluid in the abdominal cavity. **(B)** PET-CT scans showed uneven thickening of the peritoneum, omentum, and mesentery.

The patient felt an increase in abdominal circumference more than 2 months before the final chemotherapy. The enhanced CT rescan on Feb 18, 2022, suggested the following: 1) a circular line-like high-density shadow in the upper middle section, which was considered as a postoperative change; 2) a large amount of fluid in the abdominal cavity, which was new with a slightly thickened peritoneum compared with the old film from June 13, 2021; and 3) an increase in the splenic volume (SV) (**Figure 3A**). The positron emission tomography–computed tomography (PET-CT) on February 22, 2022, suggested: 1) thickening of the intestinal wall in the anastomosis area with surrounding soft tissue density shadow and a mildly increased metabolic activity in part of the intestinal wall, which was considered as a possibility of postoperative inflammatory lesions, and 2) an uneven thickening of the peritoneum, omentum, and mesentery, with peritoneal and pelvic fluid. Although metabolic activity was not increased significantly, metastasis could not be excluded (**Figure 3B**). The cancer spectrum on February 29, 2022, suggested CA19–9 of 4.3 kU/L, which was normal.

### The second surgery

Another MDT meeting was conducted, and a consensus was reached that it was necessary to perform a second surgery for the following reasons:

1) The pathology after the primary surgery suggested advanced rectal cancer, and the risk of recurrence was relatively high.
2) Laparoscopic exploration could help confirm whether it was recurrent rectal cancer and guide the subsequent strategy.
3) The patient might benefit from peritoneum, omentum, or mesentery resection plus hyperthermic intraperitoneal chemotherapy (HIPEC) for recurrent rectal cancer.

Therefore, another abdominal laparoscopic exploration was conducted on March 7, 2022. During the surgery, the ascites was yellowish and clear. Complete greater omentum resection, as well as HIPEC, was performed (**Supplementary Video 1**). During the surgery, frozen section biopsy suggested inflammatory hyperplasia. The ascites routine suggested that the ascites was yellowish and clear, with the Levantine test resulting weakly positive. There were a total of 191 cells in the ascites, of which 131 were nucleated cells, 15% were polymorphonuclear cells, and 85% were single nucleated cells. The results of the ascites biochemistry were as follows: total protein, 50 g/L; albumin, 29 g/L; globulin, 21 g/L; albumin/globulin ratio, 1.4; total bilirubin, 5.3 mmol/L; lactate dehydrogenase (LDH), 123 U/L; and adenosine deaminase (ADA), 7.4 U/L. No significant abnormalities were found in the carcinoembryonic antigen (CEA) and CA-19–9 antigen. The *Mycobacterium tuberculosis* smear and culture of the ascites was negative.

### The ascites “disappearing”

**Figure 4:**
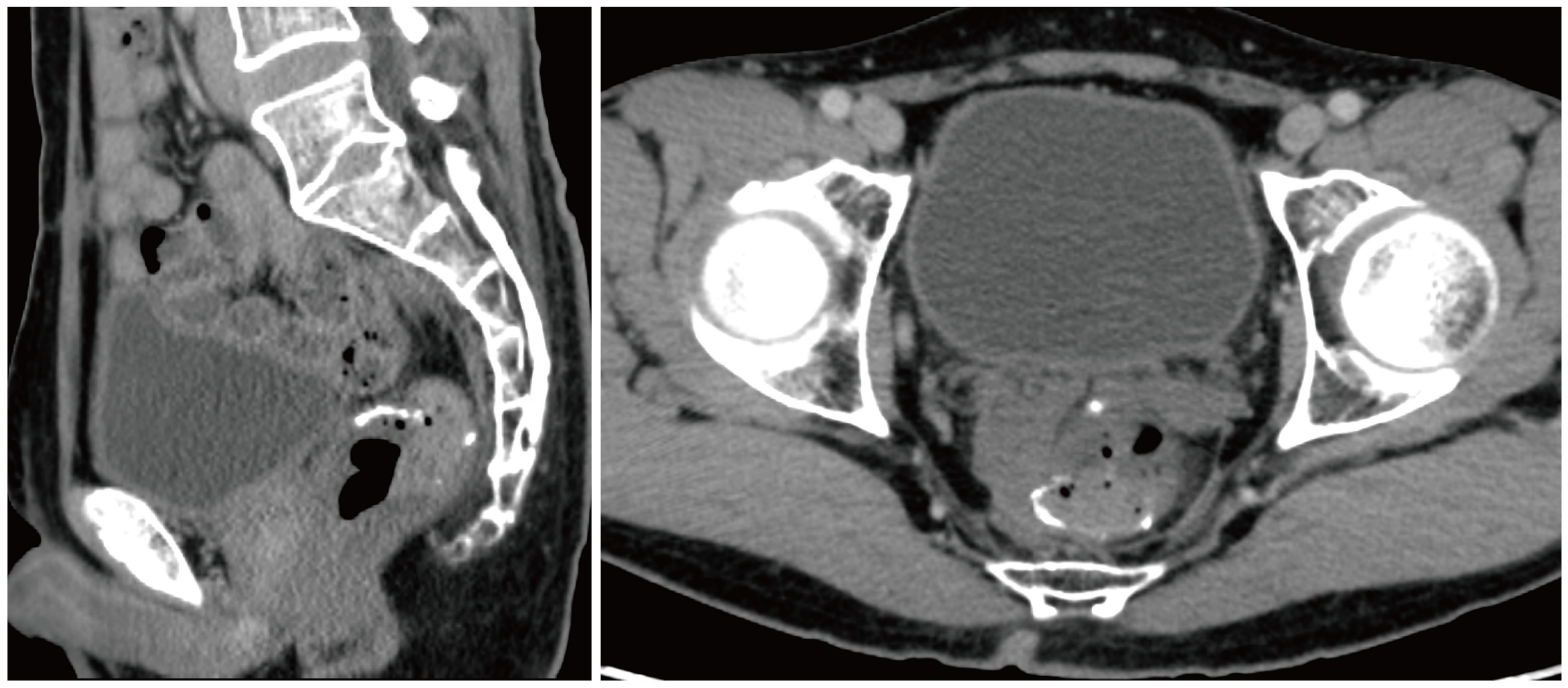
Enhanced CT examination suggested no ascites or recurrence.

However, the postoperative pathological examination suggested that there was no cancerous tissue in the omental tissue sent for examination, and local vasodilatation and congestion with fibrous tissue hyperplasia were suggested. Thus, chemotherapy was stopped after the second surgery. The latest examination was conducted on September 22, 2022, which revealed that there was no ascites or recurrence in the enhanced CT examination (**Figure 4**).

## Discussion

The 65-year-old patient in this study was diagnosed with upper rectal cancer and underwent robotic-assisted laparoscopic anterior rectal resection in our hospital. Postoperative chemotherapy using oxaliplatin and capecitabine was conducted for eight cycles; however, the patient developed ascites, which was discovered at reexamination. A second surgery was performed for HIPEC and exploration, and chemotherapy was stopped after the second surgery. Finally, ascites started disappearing until the last follow-up.

Ascites is generally classified as leaky or exudative. The ascites of this patient had a high protein content, a high cell count, and a positive Levantine test, suggesting it tended to be exudative. Exudative ascites is commonly associated with tumors, tuberculosis, and inflammatory diseases. Moreover, we believed that locally advanced rectal cancer reported intra- and postoperatively has a high prevalence of abdominopelvic implantation metastasis. When postoperative ascites occurred, after MDT discussion, tumor-related ascites was the first thought. This was the reason for the HIPEC at the second surgery. However, the second surgery revealed no recurrence after pathological examination and also ruled out tuberculosis; thus, the ascites could be caused by other factors.

In addition to an abdominopelvic ascites, the patient also developed an increased SV. A multicenter study demonstrated that more than 70% of patients with stage III colon cancer treated with an oxaliplatin-containing chemotherapy experienced an increased SV, even up to 94% when using a CapOX (capecitabine and oxaliplatin) regimen (7). It was also reported that an increased SV was significantly correlated with hepatic damage caused by oxaliplatin (8). Because the patient felt the increase in abdominal circumference more than 2 months after the final chemotherapy, it indicated that ascites occurred during chemotherapy. Therefore, we assumed that oxaliplatin might have contributed to the occurrence of ascites.

Although oxaliplatin has contributed significantly to better prognosis in patients with CRC, it has a number of serious side effects. In addition to the most common neurotoxicity and myelosuppression, ototoxicity, delayed hypersensitivity reactions, and rhabdomyolysis induced by oxaliplatin have also been reported individually (9–16). Oxaliplatin-induced damage to the liver has also been reported by Rubbia-Brandt et al. in 2004 (17), and damage to the hepatic sinusoid has been widely validated in liver tissue after treatment with oxaliplatin (18–20).

The sinusoidal damage caused by oxaliplatin could be due to the following mechanisms. Firstly, oxaliplatin could increase the porosity of the sinusoidal endothelium and stimulate the release of free radicals and the depletion of glutathione transferase, increasing the level of metalloproteinases. Secondly, the chronic hypoxia in the centrilobular region could affect nodal regenerative hyperplasia. Thirdly, oxaliplatin could damage the capillaries and parenchymal areas; the ascites produced in this manner is usually exudative, consistent with the patient’s ascites test results (20–23).

Liver injury could cause a presinusoidal increase of portal pressure due to the exudation of perisinusoidal fibrosis and the occlusion of the small vein (19). Portal hypertension typically appears years after the completion of chemotherapy and manifests clinically as varices, ascites, splenomegaly, and thrombocytopenia (24). This condition is also defined as portal–sinusoidal vascular disease (PSVD), which was proposed by the VALDIG (Vascular Liver Disease Interest Group) (25). Therefore, after excluding liver, kidney, and heart diseases, the increased SV and ascites were considered to be due to liver function damage caused by oxaliplatin.

However, the occurrence of oxaliplatin-induced ascites during adjuvant chemotherapy has not been reported previously. Hence, this is the first report of the case of a 65-year-old man with rectal cancer treated with oxaliplatin who developed significant ascites after surgery and the ascites disappearing after stopping chemotherapy.

Following 9 months after oxaliplatin withdrawal, the increased SV of the patient in this study returned to the preoperative size, and there was no reoccurrence of ascites. Previous studies also reported that half of the cases with increased SV improved to the same level as that before surgery 1 year after completing adjuvant chemotherapy, which confirmed that the splenomegaly and ascites in this case were caused by the oxaliplatin-induced hepatic impairment (26). With regard to prognosis, no relationship has been found between increased SV and disease-free survival (27). However, the prognosis and the subsequent manifestation of portal hypertension in this patient needed follow-up.

Our case report has some limitations. As this is the first time an adverse event has been identified, the report was based only on the experience of a single patient. Therefore, our conclusions may not be broadly representative, and more cases are needed for further validation. In addition, due to the lack of a control group, we were unable to compare differences in the incidence of ascites between patients using oxaliplatin and those not using oxaliplatin; thus, it was not possible to more accurately assess the relationship between oxaliplatin treatment and ascites occurrence (28). In the future, we will include more patients in order to improve the representativeness of the conclusions. At the same time, we will also strengthen the discussion on the mechanism of the relationship between oxaliplatin and ascites for a deeper understanding of its biological basis. In addition, we will enhance long-term follow-up to assess the long-term impact of this adverse event. Finally, we will consider setting up control groups to more accurately assess the relationship between oxaliplatin treatment and ascites development.

## Conclusion

The case under study suggests that chemotherapy with oxaliplatin might cause ascites. However, the exact mechanism is unclear. When patients with CRC who underwent chemotherapy with oxaliplatin develop ascites, surgeons should actively determine whether it is caused by tumor recurrence and metastasis to avoid unnecessary surgery.

## Data availability statement

The original contributions presented in the study are included in the article/**Supplementary Material**. Further inquiries can be directed to the corresponding author.

## Ethics statement

Written informed consent was obtained from the individual(s) for the publication of any potentially identifiable images or data included in this article.

## Author contributions

H-MC: Data curation, Formal analysis, Resources, Software, Writing – original draft. X-PS: Resources, Writing – review & editing. Z-QW: Supervision, Writing – review & editing. X-YW: Project administration, Supervision, Validation, Writing – review & editing.
